# Supplementary figures and images for: Integrating In Silico and In Vitro Approaches to Identify Natural Peptides with Selective Cytotoxicity against Cancer Cells
Source: Int J Mol Sci. 2024 Jun 21;25(13):6848. doi: 10.3390/ijms25136848 (PMC11240926; doi:10.3390/ijms25136848)

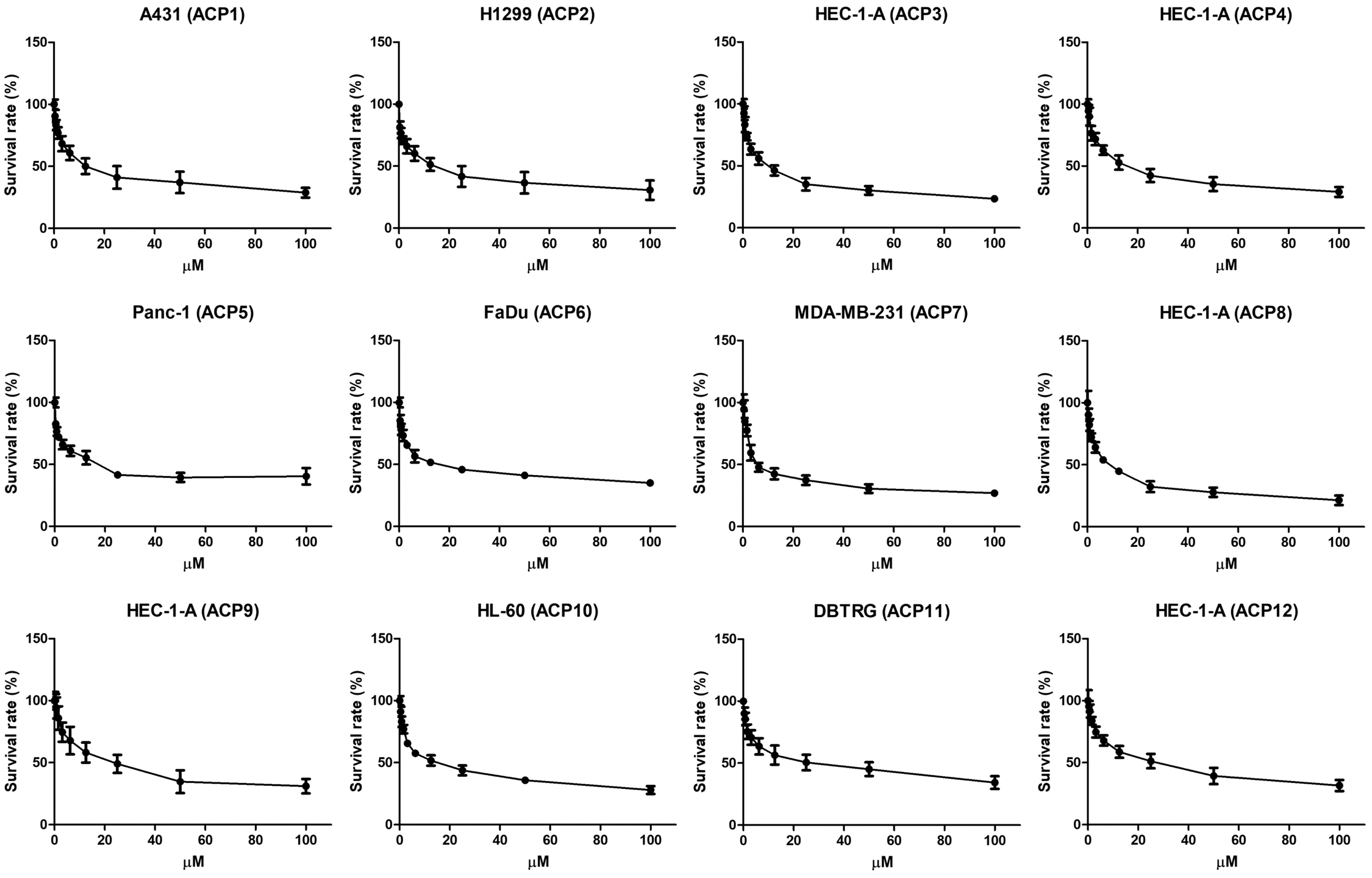

Supplement: Supplementary file 1 [file ijms-25-06848-s001.zip › IJMS_Supplementary Figure S1.png]
